# Statin Therapy and the Risk of COVID-19: A Cohort Study of the National Health Insurance Service in South Korea

**DOI:** 10.3390/jpm11020116

**Published:** 2021-02-10

**Authors:** Tak Kyu Oh, In-Ae Song, Young-Tae Jeon

**Affiliations:** 1 Department of Anesthesiology and Pain Medicine, Seoul National University Bundang Hospital, Gumi-ro 173 Beon-gil, Bundang-gu, Seongnam-si, Gyeonggi-do 13620, Korea; airohtak@hotmail.com (T.K.O.); songoficu@outlook.kr (I.-A.S.); 2 Department of Anesthesiology and Pain Medicine, Seoul National University College of Medicine, 103 Daehak-ro, Jongno-gu, Seoul 03080, Korea

**Keywords:** hydroxymethylglutaryl-CoA reductase inhibitors, infections, hospital mortality

## Abstract

We aimed to investigate whether statin therapy is associated with the incidence of coronavirus disease 2019 (COVID-19) among the South Korean population. In addition, we examined whether statin therapy affects hospital mortality among COVID-19 patients. The National Health Insurance Service (NHIS)-COVID-19 database in South Korea was used for data extraction for this population-based cohort study. A total of 122,040 adult individuals, with 22,633 (18.5%) in the statin therapy group and 101,697 (91.5%) in the control group, were included in the analysis. Among them, 7780 (6.4%) individuals were diagnosed with COVID-19 and hospital mortality occurred in 251 (3.2%) COVID-19 cases. After propensity score matching, logistic regression analysis showed that the odds of developing COVID-19 were 35% lower in the statin therapy group than in the control group (odds ratio: 0.65, 95% confidence interval: 0.60 to 0.71; *p* < 0.001). Regarding hospital mortality among COVID-19 patients, the multivariable model indicated that there were no differences between the statin therapy and control groups (odds ratio: 0.74, 95% confidence interval: 0.52 to 1.05; *p* = 0.094). Statin therapy may have potential benefits for the prevention of COVID-19 in South Korea. However, we found that statin therapy does not affect the hospital mortality of patients who are diagnosed with COVID-19.

## 1. Introduction

On 31 December 2019, 27 cases of coronavirus disease 2019 (COVID-19) with pneumonia of unknown etiology were first reported in Wuhan city, Hubei, China [1]. Thereafter, there was a COVID-19 outbreak worldwide and the World Health Organization declared the outbreak a public health emergency of international concern on 30 January 2020 [2] and a pandemic crisis on 11 March 2020 [3]. By 10 August 2020, approximately 5 million COVID-19 cases and 150,000 COVID-19-related deaths had been reported in the United States [4]. As of August 2020, no vaccine had been developed for COVID-19 [5] and it is still a public and global health crisis.

Statins, which are the most commonly prescribed drugs worldwide [6], reduce fatal cardiovascular events by lowering serum cholesterol levels [7]. More than 1 billion people are estimated to take statins globally [8]. In addition to their lipid-lowering activity, statins are known to modulate immune responses in several mechanisms, including immune cell adhesion and migration, cytokine production, and antigen presentation [9]. This is known as the “pleiotropic effect” of statins [10]. A previous meta-analysis demonstrated that statins have immunomodulatory effects in the treatment of infectious conditions such sepsis [11]. Regarding viral infections, statin therapy has been reported to reduce influenza-related deaths [12]. A recently completed randomized controlled trial (NCT02056340) indicated that patients who received atorvastatin 40 mg showed a significant improvement in the symptoms of influenza when compared to the placebo group. Similar to influenza, COVID-19 causes severe respiratory symptoms by triggering an inflammatory host response, and some immunomodulatory therapies have been reported as supportive therapies in the treatment of COVID-19 [13]. Additionally, severe acute respiratory syndrome coronavirus 2 (SARS-CoV-2), the causal agent of COVID-19, causes downregulation of angiotensin-converting enzyme 2 (ACE2), thus reducing its protective effects on various tissues. Statin is known to experimentally upregulate ACE2 via epigenetic modifications [14]. This suggests that it may be beneficial for treating patients with COVID-19. A recent retrospective review of 13,981 patients with COVID-19 showed that statin therapy is associated with lower all-cause mortality among patients with COVID-19 [15]. However, robust information regarding this finding is still lacking, and the effect of statin therapy on the prevention of COVID-19 has not yet been identified.

Therefore, we aimed to investigate whether statin therapy is associated with the incidence of COVID-19 among the South Korean population by using the National Health Insurance Service (NHIS)-COVID-19 cohort database. Additionally, we examined whether statin therapy affects hospital mortality among patients diagnosed with COVID-19 in South Korea.

## 2. Materials and Methods

### 2.1. Study Design and Ethical Statement

As a population-based cohort study, this study was conducted according to the Reporting of Observational Studies in Epidemiology guidelines [16]. The study protocol was approved by the Institutional Review Board of Seoul National University Bundang Hospital (X-2004-604-905) and the Health Insurance Review and Assessment Service (NHIS-2020-1-291). Informed consent was waived because data analyses were performed retrospectively using anonymized data derived from the South Korean NHIS database.

### 2.2. NHIS-COVID-19 Cohort Database and Study Population

The NHIS-COVID-19 cohort database was developed for medical research through cooperation between the NHIS and the Korea Centers for Disease Control and Prevention (KCDC). The KCDC provides information on patients who were diagnosed with COVID-19 from 1 January 2020 to 4 June 2020. The polymerase chain reaction (PCR) method was used for COVID-19 detection in South Korea. The NHIS COVID-19 database included all patients with COVID-19 who were confirmed as positive according to the COVID-19 test, regardless of their hospitalization status. Therefore, patients with COVID-19 who were admitted to the hospital with severe symptoms, as well as those with no or mild symptoms, were included in the database. In South Korea, patients who were diagnosed with COVID-19 were admitted to the hospital if they had severe symptoms, such as pneumonia. Conversely, if they had mild or no symptoms, they were isolated and closely monitored in certain government-managed centers. The information included the confirmation date of the COVID-19 diagnosis, the results of the treatment and the demographic information of each patient. COVID-19 patients who are undergoing treatment in the hospital are not included in this database because their treatment results have not yet been determined. Using the information provided by the KCDC, the NHIS extracted the control population by using stratification methods to categorize the COVID-19 patients according to their age, sex, and place of residence in February 2020.

In the NHIS-COVID-19 cohort database, all disease diagnoses are classified according to the International Classification of Diseases (ICD)-10 codes. Prescription information concerning drugs and/or procedures from 2015 to 2020 was also included. For the present study, data were extracted on 26 June 2020 by an independent medical record technician at the NHIS center who was unaffiliated with this study. Individuals who were ≥ 20 years old were included in the NHIS-COVID-19 cohort database.

### 2.3. Statin Users (Exposure Group)

The prescription information each individual recorded from 2019 to 2020 was extracted. Statin users were defined as those who had been prescribed continuous oral statin over a period of ≥ 30 days and the control group was defined as all other subjects. In this study, statin users took statins after hospitalization for the treatment of COVID-19.

### 2.4. Endpoints

The primary endpoint of this study was the diagnosis of COVID-19 and the duration of evaluation was from 1 January 2020 to 4 June 2020. The secondary endpoint of this study was hospital mortality amongst patients who were diagnosed with COVID-19.

### 2.5. Confounders

The following data were collected as confounders: (1) demographic information (age and sex); (2) place of residence (Seoul, Gyeonggi-do, Daegu, Gyeongsangbuk-do, and Other areas); (3) underlying disability; (4) income level in 2020; (5) the Charlson comorbidity index, which was calculated from 1 January 2019 to 31 December 2019 based on registered ICD-10 diagnostic codes (Table S1); and (6) other use of cardiovascular medications (aspirin, clopidogrel, angiotensin-converting enzyme inhibitors, angiotensin II receptor blockers, calcium channel blockers, and beta-blockers). Patients were categorized into seven groups according to age: 20–29 years, 30–39 years, 40–49 years, 50–59 years, 60–69 years, 70–79 years and ≥80 years.

### 2.6. Statistical Analyses

The baseline characteristics of the participants in this study are presented as numbers with percentages for categorical variables and as mean values with standard deviations for continuous variables. Firstly, we performed propensity score matching to reduce confounders in observational studies by using the nearest neighbor method with a 1:1 ratio without replacement and a caliper width of 0.1 [17]. Logistic regression analysis was performed to calculate propensity scores as a logistic model and all covariates were included in the propensity score model. The absolute value of the standardized mean difference (ASD) was used to determine the balance between the statin therapy group and the control group before and after propensity score matching. ASDs between the two groups were set below 0.1 to determine whether the two groups were well balanced through propensity score matching. After confirming a good balance between the two groups, we performed a univariable logistic regression analysis for the development of COVID-19 in the propensity score-matched cohort. Secondly, as a sensitivity analysis, we performed multivariable logistic regression analysis for the development of COVID-19 in the entire cohort to determine whether the results obtained from the propensity score-matched cohort were generalizable to the entire cohort. Furthermore, we investigated the risk of the development of COVID-19 in the statin therapy group with other covariates in context, rather than in isolation. All covariates were included in the multivariable model for adjustment. The Charlson comorbidity index was excluded in the model to avoid multicollinearity of the underlying comorbidities, which were used to calculate the Charlson comorbidity index.

Finally, we constructed a multivariable logistic model for hospital mortality among patients who were diagnosed with COVID-19 to investigate whether statin therapy affected their mortality when compared to the control group. Hosmer–Lemeshow statistics were used to confirm that the goodness of fit of multivariable models was *p* > 0.05. We confirmed that there was no multicollinearity in any of the multivariable models across the entire cohort; the variance inflation factor was < 2.0. The results of the logistic regression models are presented as odds ratios (ORs) with 95% confidence intervals (CIs). A receiver–operator characteristic (ROC) analysis was performed to validate the use of logistic regression analysis for this study. R software (version 3.6.3; R Foundation for Statistical Computing, Vienna, Austria) and SAS software version 9.4 (SAS Institute Inc., Cary, NC, USA) were used for all the analyses and *p* < 0.05 was considered statistically significant.

## 3. Results

### 3.1. Study Population

**Figure 1:**
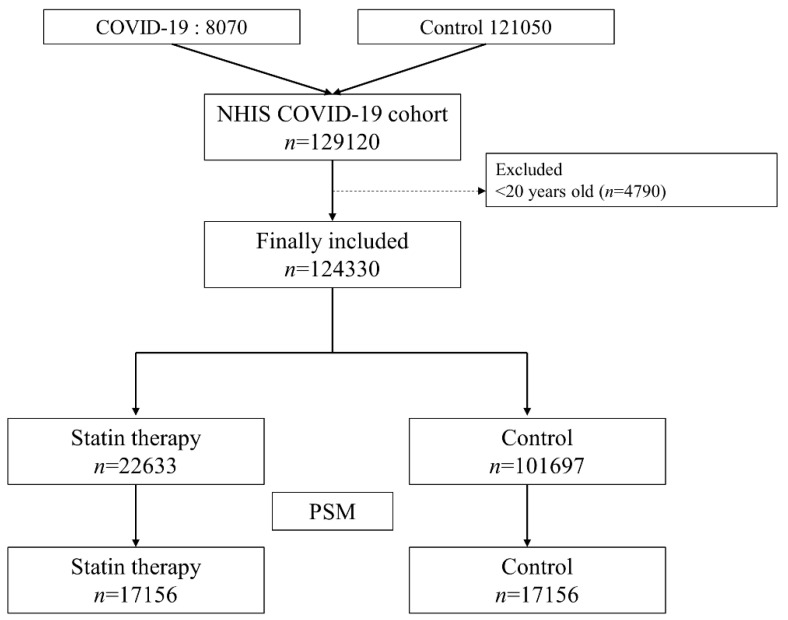
Flow chart depicting the process of participant selection. COVID-19, coronavirus disease-2019; PSM, propensity score matching.

At the data extraction date (26 June 2020), the NHIS COVID-19 cohort was comprised of 8070 COVID-19 patients and 121,050 controls. Among them, 4790 individuals who were <20 years old were excluded from the analysis. Thus, 122,040 adults were included in the analysis; 22,633 individuals (18.5%) were in the statin therapy group and 101,697 individuals (91.5%) were in the control group. A total of 7780 (6.4%) individuals were diagnosed with COVID-19 during the study period and hospital mortality occurred in 251 (3.2%) COVID-19 cases. After propensity score matching, 34,312 individuals (17,156 individuals in each group) were included in the final analysis. The results of the comparison of characteristics between the statin therapy group and the control group are presented in Table 1. All ASDs were <0.1, indicating that all covariates between the two groups were well-balanced as a result of propensity score matching. The patient selection flowchart is presented in Figure 1.

### 3.2. Risk of COVID-19 in South Korea

Table 2 shows the development of COVID-19 among the South Korean population before and after propensity score matching. After propensity matching, 938 of the 17,156 (5.5%) individuals in the statin therapy group were diagnosed with COVID-19, whereas 1395 of the 17,156 (8.1%) individuals in the control group were diagnosed with COVID-19. Logistic regression analysis after propensity score matching showed that the odds of developing COVID-19 were 35% lower in the statin therapy group than in the control group (OR: 0.65, 95% CI: 0.60 to 0.71; *p* < 0.001). Table 3 shows the results of the multivariable logistic regression model for the development of COVID-19 across the entire cohort. In the multivariable model, the incidence of COVID-19 in the statin therapy group was 41% lower than that in the control group (OR, 0.59; 95% CI, 0.55. 0.64; *p* < 0.001). Hosmer–Lemeshow statistics showed that the goodness of fit was appropriate in the models (*p* > 0.05), and the area under curve (AUC) of the multivariable models in the ROC analyses was 0.81 (95% CI: 0.80 to 0.81).

### 3.3. Hospital Mortality among COVID-19 Patients

Table 4 shows the results of the multivariable logistic regression model for hospital mortality in COVID-19 patients. In the multivariable model, there were no differences between the statin therapy group and the control group in terms of hospital mortality (OR: 0.74, 95% CI: 0.52 to 1.05; *p* = 0.094). Hosmer–Lemeshow statistics showed that the goodness of fit was appropriate in the three models (*p* > 0.05), and the AUC of the multivariable model in the ROC analysis was 0.83 (95% CI: 0.82 to 0.83).

## 4. Discussion

In this study, we investigated whether statin therapy is associated with the incidence of COVID-19 among the general South Korean population and examined whether statin therapy affects hospital mortality among COVID-19 patients. Our results show that statin therapy is associated with a lower risk of COVID-19 among the South Korean population. However, we did not find an association between statin therapy and a reduction in mortality among COVID-19 patients. This result is in contrast to that of a recent retrospective observational study [15]. To the best of our knowledge, this is the first study to show that statin therapy may have a potential benefit in lowering the risk of COVID-19 in the general population. Considering that a recent review reported that dyslipidemia increases the risk of COVID-19 infection [18], greater attention should be paid to statin therapy as it may lower the risk of COVID-19.

To interpret our results, the effect of statin therapy on the immune system should be considered. Based on the immunomodulatory effects of statins, statin therapy was considered as a prophylactic treatment option in the pandemic crisis of influenza in the past [19]. This is supported by the fact that when the influenza virus enters a human host, the replication and transportation of its own viral components occur via intracellular pathways [20,21]. Thus, the anti-inflammatory and immunomodulatory effects of statins can be effective because statins block downstream molecules, which are the main factors that influence virus infectivity [22]. David S Fedson insisted in 2009 that if another influenza pandemic occurs, anti-inflammatory and immunomodulatory agents such as statins would be beneficial for prophylaxis [23]. SARS-CoV-1 causes an intense proinflammatory host response similar to that of the influenza virus. Therefore, anti-inflammatory and immunomodulatory agents may also be beneficial for prophylaxis in COVID-19 cases [24].

The relationship between ACE2 and statin therapy is another important issue that should be considered. Statin interferes with ACE2 signaling [24] and it decreases the activity of SARS-CoV-2. Once SARS-CoV-2 enters the cell, it causes ACE2 downregulation, thus reducing its protective effect against the infection of various tissues [13]. It has been reported that the downregulation of ACE2 by SARS-CoV-2 leads to organ injury in COVID-19 patients [13]. Therefore, the upregulation of ACE2 induced by statins via epigenetic modifications has a beneficial effect on COVID-19 patients [14]. However, our study also showed that the use of an ACE2 inhibitor is not associated with the incidence of COVID-19 and hospital mortality among COVID-19 patients. The effect of ACE2 on the prevention and outcomes of COVID-19 remains controversial. Thus, further studies are needed to clarify this in the future.

We did not find any significant association between statin therapy and hospital mortality among COVID-19 patients. However, a recent retrospective observational study reported that statin therapy is associated with lower mortality among COVID-19 patients [15]. The sample size of our study was smaller (7780) than that of this previous study (13,981). Although both studies adjusted for the underlying comorbidities of the cohort, we did not include details of the disease severity in the COVID-19 patients in the present study, whereas the previous study included laboratory findings upon admission to the hospital. Therefore, the results of our study should be carefully interpreted. Although the results of both studies suggest that the effect of statin therapy on mortality remains controversial, information is still lacking on this issue.

This study has some limitations. Firstly, some important variables, including body mass index and lifestyle factors such as history of smoking and alcohol consumption, were not included in the analysis because they were not available in the NHIS database. Secondly, both propensity score modeling and multivariable adjustment are known to reduce known and measured confounders. Therefore, there might be some residual confounders. Thirdly, to calculate the Charlson comorbidity index, we defined the comorbidities using ICD-10 codes. However, the diseases that are specified by the ICD-10 codes may differ from the actual underlying diseases in our study population. Furthermore, our analysis was based on statin prescription data in the NHIS database—we did not assess compliance among those classified as statin users. Fourthly, we did not consider the type and daily dosage of statin because the NHIS database provides the prescription information with masking of the type and dosage. As daily dosage and type of statin may affect the results, these effects should be evaluated in future studies. Lastly, the symptoms at the time of diagnosis of COVID-19 were not reflected in this study because we included all patients with COVID-19 who were diagnosed according to the PCR test. As the clinical meaning could be very different between asymptomatic and symptomatic patients with COVID-19, it might be a limitation of this study.

## 5. Conclusions

Using the NHIS-COVID-19 database cohort, we showed that statin therapy may have potential benefits for the prevention of COVID-19 in South Korea. However, we also found that it did not affect the hospital mortality of patients who were diagnosed with COVID-19. Considering its inexpensive price, safety and popularity, statin therapy should be considered as a prophylactic treatment in the COVID-19 pandemic.

## Figures and Tables

**Table 1:** Comparison of characteristics between statin users and the control group before and after propensity score matching.

	Before Propensity Score Matching (*n* = 124,330)			After Propensity Score Matching (*n* = 34,312)		
	Statin User *n* = 22,633	Control Group *n* = 101,697	ASD	Statin User *n* = 17,156	Control Group *n* = 17,156	ASD
Age, year						
20–29	146 (0.6)	32,234 (31.7)		146 (0.9)	179 (1.0)	
30–39	331 (1.5)	12,823 (12.6)	0.929	329 (1.9)	220 (1.3)	0.053
40–49	1222 (5.4)	15,297 (15.0)	0.427	1176 (6.9)	854 (5.0)	0.083
50–59	5513 (24.4)	19,747 (19.4)	0.115	4768 (27.8)	4786 (27.9)	0.002
60–69	7640 (33.8)	12,029 (11.8)	0.463	5503 (32.1)	5820 (33.9)	0.039
70–79	4965 (21.9)	5461 (5.4)	0.400	3232 (18.8)	3221 (18.8)	0.002
≥80	2816 (12.4)	4106 (4.0)	0.255	2002 (11.7)	2076 (12.1)	0.013
Sex, male	8147 (36.0)	40,579 (39.9)	0.081	6141 (35.8)	6263 (36.5)	0.015
Residence						
Seoul	1001 (4.4)	7055 (6.9)		788 (4.6)	778 (4.5)	
Gyeonggi-do	15,533 (68.6)	65,960 (64.9)	0.081	11,654 (67.9)	11,603 (67.6)	0.006
Daegu	1111 (4.9)	5744 (5.6)	0.034	839 (4.9)	845 (4.9)	0.002
Gyeongsangbuk-do	3242 (14.3)	11,865 (11.7)	0.076	2488 (14.5)	2551 (14.9)	0.011
Other area	1746 (7.7)	11,073 (10.9)	0.119	1387 (8.1)	1379 (8.0)	0.002
Underlying disability	2687 (11.9)	4968 (4.9)	0.216	1836 (10.7)	1913 (11.2)	0.014
Income level						
Q1 (Lowest)	5999 (26.5)	26,388 (25.9)		4490 (26.2)	4481 (26.1)	
Q2	3496 (15.4)	21,483 (21.1)	0.157	2782 (16.2)	2773 (16.2)	0.002
Q3	4887 (21.6)	22,887 (22.5)	0.022	3732 (21.8)	3694 (21.5)	0.005
Q4 (Highest)	7892 (34.9)	29,177 (28.7)	0.130	5886 (34.3)	5936 (34.6)	0.006
Unknown	359 (1.6)	1762 (1.7)	0.012	266 (1.6)	272 (1.6)	0.003
Charlson comorbidity index 2020	3.6 (3.4)	1.4 (2.6)	0.640	3.2 (3.2)	3.1 (3.3)	0.018
Hypertension	15,826 (69.9)	16,901 (16.6)	1.162	10,663 (62.2)	11,185 (65.2)	0.066
DM without chronic complication	8099 (35.8)	5682 (5.6)	0.630	4668 (27.2)	4403 (25.7)	0.032
DM with chronic complication	2944 (13.0)	1311 (1.3)	0.348	1560 (9.1)	1171 (6.8)	0.067
Peripheral vascular disease	3901 (17.2)	3297 (3.2)	0.371	2446 (14.3)	2190 (12.8)	0.039
Renal disease	733 (3.2)	659 (0.6)	0.146	438 (2.6)	415 (2.4)	0.008
Rheumatic disease	1097 (4.8)	1861 (1.8)	0.141	699 (4.1)	790 (4.6)	0.025
Dementia	1684 (7.4)	2242 (2.2)	0.200	1168 (6.8)	1239 (7.2)	0.016
Peptic ulcer disease	3331 (14.7)	6541 (6.4)	0.233	2345 (13.7)	2334 (13.6)	0.002
Hemiplegia or paraplegia	204 (0.9)	364 (0.4)	0.058	150 (0.9)	147 (0.9)	0.002
Moderate or severe liver disease	43 (0.2)	103 (0.1)	0.020	34 (0.2)	35 (0.2)	0.001
Mild liver disease	6117 (27.0)	7495 (7.4)	0.442	3967 (23.1)	3975 (23.2)	0.001
Chronic pulmonary disease	4303 (19.0)	9813 (9.6)	0.239	3027 (17.6)	3059 (17.8)	0.005
Cerebrovascular disease	3447 (15.2)	2316 (2.3)	0.360	1971 (11.5)	1651 (9.6)	0.052
Congestive heart failure	2079 (9.2)	1604 (1.6)	0.263	1241 (7.2)	1077 (6.3)	0.033
Myocardial infarction	722 (3.2)	465 (0.5)	0.156	329 (1.9)	232 (1.4)	0.032
Malignancy	6157 (27.2)	15,856 (15.6)	0.261	4355 (25.4)	4439 (25.9)	0.011
Metastatic solid tumor	1350 (6.0)	2722 (2.7)	0.139	963 (5.6)	1049 (6.1)	0.021
AIDS/HIV	2 (0.0)	30 (0.0)	0.022	2 (0.0)	4 (0.0)	0.012
Other cardiovascular drug use						
Aspirin	4845 (21.4)	2009 (2.0)	0.474	2468 (14.4)	1862 (10.9)	0.086
Clopidogrel	2874 (12.7)	823 (0.8)	0.357	1353 (7.9)	797 (4.6)	0.097
ACEi	575 (2.5)	191 (0.2)	0.150	272 (1.6)	171 (1.0)	0.037
ARB	9836 (43.5)	8020 (7.9)	0.718	6285 (36.6)	6390 (37.2)	0.012
CCB	4954 (21.9)	3687 (3.6)	0.442	3094 (18.0)	2983 (17.4)	0.016
Beta blocker	2203 (9.7)	1206 (1.2)	0.288	1189 (6.9)	997 (5.8)	0.038

Presented as a number with percentage or mean value with standard deviation. ASD, absolute value of standardized mean difference; DM, diabetes mellitus; AIDS, acquired immune deficiency syndrome; HIV, Human Immunodeficiency Virus; ACEi, angiotensin-converting enzyme inhibitors; ARB, angiotensin II receptor blockers; CCB, calcium channel blocker.

**Table 2:** Development of COVID-19 before and after PSM.

Variable	Development of COVID-19	Logistic Regression Analysis	*p*-Value
		OR (95% CI)	
Before PSM			
Control group	6471/101,697 (6.4)	1	
Statin user	1309/22,633 (5.8)	0.90 (0.85, 0.96)	0.0011
After PSM			
Control group	1395/17,156 (8.1)	1	
Statin user	938/17,156 (5.5)	0.65 (0.60 0.71)	<0.001

Presented as a number with percentage. PSM, propensity score matching; OR, odds ratio; CI, confidence interval.

**Table 3:** Multivariable logistic regression model for diagnosis of COVID-19 in the entire National Health Insurance Service (NHIS)-COVID-19 cohort.

Variable	Multivariable Model	*p*-Value
	OR (95% CI)	
Statin users (vs control group)	0.59 (0.55, 0.64)	<0.001
Age, year		
20–29	1	
30–39	0.91 (0.84, 1.00)	0.038
40–49	0.86 (0.79, 0.93)	<0.001
50–59	0.77 (0.72, 0.83)	<0.001
60–69	0.63 (0.58, 0.69)	<0.001
70–79	0.46 (0.41, 0.52)	<0.001
≥80	0.35 (0.30, 0.40)	<0.001
Income level		
Q1	1	
Q2	0.80 (0.75, 0.86)	<0.001
Q3	0.78 (0.73, 0.84)	<0.001
Q4	0.84 (0.79, 0.90)	<0.001
Unknown	0.77 (0.63, 0.94)	0.009
Sex, male	1.03 (0.98, 1.08)	0.227
Residence		
Seoul	1	
Gyeonggi-do	0.92 (0.83, 1.01)	0.078
Daegu	0.98 (0.86, 1.13)	0.789
Gyeongsangbuk-do	0.89 (0.80, 1.01)	0.062
Other area	0.88 (0.78, 0.99)	0.031
Underlying disability	1.04 (0.94, 1.15)	0.430
Underlying comorbidities		
Hypertension	1.04 (0.95, 1.13)	0.406
DM without chronic complication	1.94 (1.80, 2.09)	<0.001
DM with chronic complication	0.89 (0.78, 1.02)	0.101
Peripheral vascular disease	0.79 (0.71, 0.88)	<0.001
Renal disease	0.89 (0.72, 1.09)	0.250
Rheumatic disease	0.94 (0.82, 1.08)	0.387
Dementia	1.83 (1.61, 2.09)	<0.001
Peptic ulcer disease	1.26 (1.17, 1.36)	<0.001
Hemiplegia or paraplegia	2.23 (1.72, 2.87)	<0.001
Moderate or severe liver disease	0.46 (0.25, 0.85)	0.013
Mild liver disease	1.99 (1.86, 2.14)	<0.001
Chronic pulmonary disease	3.63 (3.42, 3.84)	<0.001
Cerebrovascular disease	1.16 (1.03, 1.32)	0.017
Congestive heart failure	2.89 (2.60, 3.23)	<0.001
Myocardial infarction	6.43 (5.49, 7.54)	<0.001
Malignancy	1.71 (1.62, 1.81)	<0.001
Metastatic solid tumor	0.82 (0.73, 0.93)	0.003
AIDS/HIV	2.99 (1.27, 7.05)	0.012
Other cardiovascular drug use		
Aspirin	0.75 (0.66, 0.86)	<0.001
Clopidogrel	0.70 (0.59, 0.83)	<0.001
ACEi	0.80 (0.60, 1.08)	0.145
ARB	0.81 (0.74, 0.89)	<0.001
CCB	0.93 (0.83, 1.04)	0.225
Beta blocker	0.59 (0.49, 0.71)	<0.001

AUC, 0.81 (95% CI: 0.80, 0.81). OR, odds ratio; CI, confidence interval; DM, diabetes mellitus; AIDS, acquired immune deficiency syndrome; HIV, Human Immunodeficiency Virus; ACEi, angiotensin-converting enzyme inhibitors; ARB, angiotensin II receptor blockers; CCB, calcium channel blocker; AUC, area under curve.

**Table 4:** Multivariable logistic regression model for hospital mortality in COVID-19 patients (*n* = 7780, death = 251, 3.2%).

Variable	Multivariable Model	*p*-Value
	OR (95% CI)	
Statin users (vs control group)	0.74 (0.52, 1.05)	0.094
Age, 10 year increase	2.95 (2.48, 3.50)	<0.001
Income level 2020 (Feb)		
Q1 (Lowest)	1	
Q2	0.95 (0.58, 1.55)	0.834
Q3	1.09 (0.71, 1.67)	0.698
Q4 (highest)	0.91 (0.63, 1.31)	0.605
Unknown	0.54 (0.14, 2.10)	0.374
Sex, male	2.23 (1.65, 3.03)	<0.001
Residence at Februrary, 2020		
Seoul	1	
Gyeonggi-do	2.10 (0.68, 6.45)	0.196
Daegu	2.96 (0.84, 10.46)	0.092
Gyeongsangbuk-do	2.17 (0.68, 6.89)	0.190
Other area	1.60 (0.47, 5.52)	0.455
Underlying disability	1.25 (0.88, 1.78)	0.207
Underlying comorbidities		
Hypertension	1.69 (1.08, 2.63)	0.021
DM without chronic complication	1.86 (1.34, 2.57)	<0.001
DM with chronic complication	1.66 (1.09, 2.54)	0.019
Peripheral vascular disease	1.10 (0.74, 1.63)	0.632
Renal disease	1.59 (0.95, 2.65)	0.076
Rheumatic disease	0.60 (0.31, 1.14)	0.118
Dementia	1.55 (1.07, 2.25)	0.021
Peptic ulcer disease	1.05 (0.74, 1.51)	0.773
Hemiplegia or paraplegia	2.07 (1.11, 3.86)	0.023
Moderate or severe liver disease	5.23 (1.32, 20.75)	0.019
Mild liver disease	0.79 (0.58, 1.09)	0.148
Chronic pulmonary disease	1.81 (1.33, 2.47)	<0.001
Cerebrovascular disease	0.64 (0.42, 0.98)	0.042
Congestive heart failure	1.80 (1.30, 2.51)	<0.001
Myocardial infarction	0.84 (0.50, 1.41)	0.506
Malignancy	0.96 (0.70, 1.32)	0.816
Metastatic solid tumor	1.60 1.03 2.47	0.035
AIDS/HIV	1.12 0.08 15.23	0.933
Other cardiovascular drug use		
Aspirin	1.06 (0.70, 1.58)	0.792
Clopidogrel	1.36 (0.83, 2.24)	0.220
ACEi	1.22 (0.55, 2.73)	0.620
ARB	0.82 (0.58, 1.16)	0.263
CCB	0.55 (0.36, 0.84)	0.006
Beta blocker	1.52 (0.84, 2.76)	0.170

AUC: 0.83 (95% CI: 0.82, 0.83). OR, odds ratio; CI, confidence interval; DM, diabetes mellitus; AIDS, acquired immune deficiency syndrome; HIV, Human Immunodeficiency Virus; ACEi, angiotensin-converting enzyme inhibitors; ARB, angiotensin II receptor blockers; CCB, calcium channel blocker; AUC, area under curve.

## Data Availability

In-Ae Song takes responsibility for (is the guarantor of) the content of the manuscript, including the data and analysis, and the data will be available upon reasonable request to corresponding author.

## Supplementary Materials

The following are available online at https://www.mdpi.com/2075-4426/11/2/116/s1, Table S1. ICD-10 codes.
